# Hematopoietic Stem Cells and Their Niche in Bone Marrow

**DOI:** 10.3390/ijms25136837

**Published:** 2024-06-21

**Authors:** Munju Kwon, Byoung Soo Kim, Sik Yoon, Sae-Ock Oh, Dongjun Lee

**Affiliations:** 1 Department of Convergence Medicine, School of Medicine, Pusan National University, Yangsan 50612, Republic of Korea; gmj0226@naver.com; 2 School of Biomedical Convergence Engineering, Pusan National University, Yangsan 50612, Republic of Korea; bskim7@pusan.ac.kr; 3 Department of Anatomy, School of Medicine, Pusan National University, Yangsan 50612, Republic of Korea; sikyoon@pusan.ac.kr (S.Y.); hedgehog@pusan.ac.kr (S.-O.O.); 4 Transplantation Research Center, Research Institute for Convergence of Biomedical Science and Technology, Pusan National University Yangsan Hospital, Yangsan 50612, Republic of Korea

**Keywords:** hematopoietic stem cells, hematopoietic progenitor cells, bone marrow microenvironment, niche

## Abstract

Extensive research has explored the functional correlation between stem cells and progenitor cells, particularly in blood. Hematopoietic stem cells (HSCs) can self-renew and regenerate tissues within the bone marrow, while stromal cells regulate tissue function. Recent studies have validated the role of mammalian stem cells within specific environments, providing initial empirical proof of this functional phenomenon. The interaction between bone and blood has always been vital to the function of the human body. It was initially proposed that during evolution, mammalian stem cells formed a complex relationship with the surrounding microenvironment, known as the niche. Researchers are currently debating the significance of molecular-level data to identify individual stromal cell types due to incomplete stromal cell mapping. Obtaining these data can help determine the specific activities of HSCs in bone marrow. This review summarizes key topics from previous studies on HSCs and their environment, discussing current and developing concepts related to HSCs and their niche in the bone marrow.

## 1. Introduction

**Figure 1:**
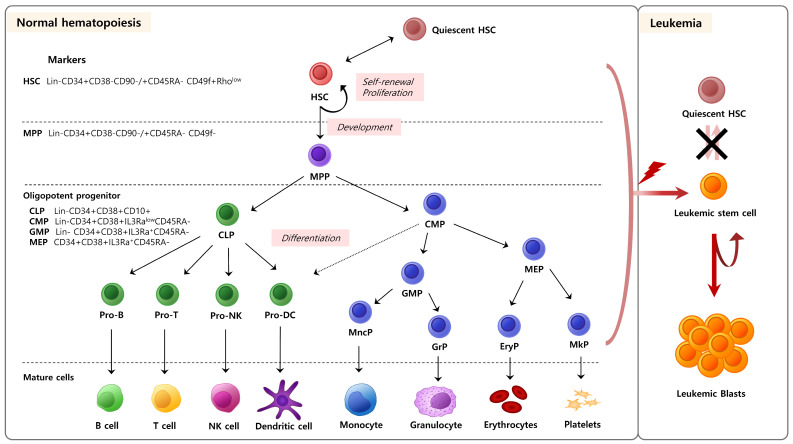
Hematopoietic stem cell (HSC) regulation in steady-state and hematological malignancies. This image shows the features of HSC regulation between normal conditions and hematological malignancy. In normal hematopoiesis, HSCs are activated in response to signals from the bone marrow microenvironment. Upon activation, HSCs undergo proliferation to increase their numbers and develop into multipotent progenitors (MPPs). MMPs can evolve into more committed lymphoid/myeloid progenitors and their respective sub-progenitors (e.g., GMP, MEP, etc.). These progenitor cells undergo further differentiation and maturation to give rise to the diverse range of blood cell types found in circulation. Each cell in the hematopoietic process can be distinguished by differentiation markers. This tightly regulated process of activation, proliferation, and differentiation ensures the continuous replenishment of blood cells to maintain homeostasis. When the HSCs and the progenitors within the developing HSCs become damaged, they can transform into leukemic stem cells (LSCs). LSCs possess self-renewal capabilities and aberrant differentiation, giving rise to leukemic blasts that result in leukemia. CLP: Common lymphoid progenitor. CMP: Common myeloid progenitor. GMP: Granulocyte–Macrophage progenitor. MEP: Megakaryocyte–erythrocyte progenitor. Pro-B: Progenitor cell-B. Pro-T: Progenitor cell-T. Pro-NK: Progenitor cell-NK. Pro-DC: Dendritic progenitor cell. MncP: Monocyte progenitor. GrP: Granulocytic progenitor. EryP: Erythrocytic progenitor. MkP: Megakaryocyte progenitor. NK cells: Natural killer cells.

**Figure 2:**
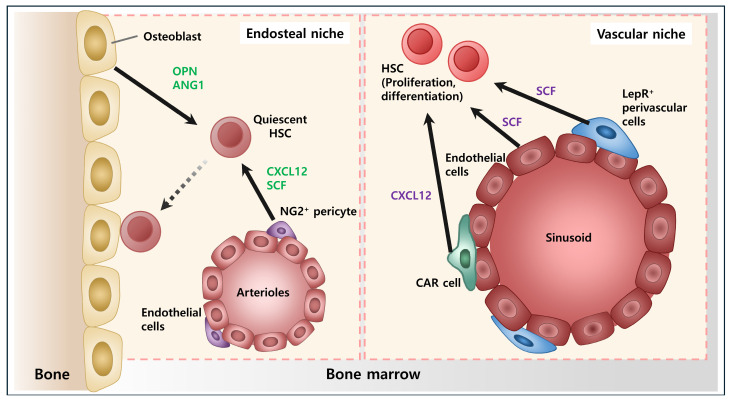
An image showing bone marrow microenvironment with their components. It shows two BM niches, two bone marrow niches, and the endosteal and vascular niches. The endosteal niche and vascular niche are two crucial microenvironments within the BM. The endosteal niche, located near the bone surface, provides a specialized environment for hematopoietic stem cells (HSCs) to reside and self-renew. The osteoblast is considered the most important cell in the endosteal niche; hence, it is also referred to as the osteoblastic niche. In contrast, the vascular niche, adjacent to blood vessels, supports HSCs by supplying nutrients and signaling molecules necessary for their proliferation and differentiation. It is composed of endothelial cells lining the blood vessels, as well as pericytes and smooth muscle cells surrounding them. Together, these niches play integral roles in regulating the maintenance and function of HSCs in the bone marrow. CAR cell: CXCL12-abundant reticular cell. OPN: Osteopontin. ANG1: Angiopoietin-1, SCF: Stem cell factor.

Blood is a bodily fluid that delivers oxygen and nutrients to cells while collecting and transporting carbon dioxide and waste products produced by cellular metabolism [1]. Blood consists of plasma (a liquid component), red blood cells, white blood cells, and platelets. Hematopoiesis is the biological process through which blood and immune cells are produced [2] (Figure 1). Hematopoietic stem cells (HSCs) in the bone marrow are responsible for continuously replenishing these cells due to their limited lifespan [3]. HSCs occupy the highest position in the hierarchy of hematopoietic cells. The HSC niche in bone marrow is a specialized microenvironment that regulates the maintenance and activity of HSCs [4]. This niche governs self-renewal and differentiation of HSCs, ensuring the continual maintenance of hematopoiesis [5]. The bone marrow microenvironment was first introduced as a niche for HSCs in the 1970s [6]. The niche supplies the necessary components for the self-renewal and differentiation of HSCs. Additionally, the niche controls the states of rest and progression at various stages of the cell cycle in stem cells [6] (Figure 2). It also communicates crucial information to stem cells regarding the surrounding tissue, influences the development of stem cell offspring, and helps prevent genetic mutations [7]. Numerous studies have revealed the significance of HSCs and their niche, leading to a better understanding of their relationship [7,8,9,10,11].

Due to global advancements in aging research and the increase in life expectancy over the past 150 years, studies on the physiological changes that occur in organisms as they age have made substantial progress [12,13]. Aging is characterized by a progressive decline in the function of many organs and tissues that, in some cases, can contribute to the development of cancer [14]. The hematopoietic system undergoes alterations with age, which affects the performance and number of HSCs and the composition of blood cells [15], increasing the likelihood of acquiring age-related blood illnesses such as anemia, a weakened immune response, and blood cancer. After a defined period, blood cells undergo differentiation and maturation and are eventually destroyed, preserving the equilibrium state. Hematological disorders are medical ailments characterized by an imbalance in homeostasis [10]. Hematopoietic tissue cancer (blood cancer) is a malignancy that originates in bone marrow [8] and is characterized by the excessive growth of abnormal blood cells [16]. These disorders are due to abnormalities in HSCs, the initiating cells in the hematopoietic system. Therefore, targeting only specific cells while minimizing damage to normal cells remains challenging [17,18]. Consequently, stem cell therapy is emerging as a promising alternative for treating hematological diseases, including those related to aging.

Stem cell therapy is highly regarded for its potential in treating not only blood-related diseases but also for regenerating damaged tissues and organs. Stem cells used in related research encompass various types, including adult stem cells such as HSCs and mesenchymal stem cells (MSCs), embryonic stem cells (ESCs), and induced pluripotent stem cells (iPSCs) created by reprogramming somatic cells back to a pluripotent state [19,20]. MSCs, being multipotent stromal cells, exhibit the capacity to differentiate into a variety of cell types, including bone, cartilage, and adipocytes [20,21,22,23]. Consequently, numerous research findings have suggested their therapeutic potential in diverse diseases such as cartilage regeneration [24,25] and neurological disease recovery [25,26,27,28]. ESCs present immense therapeutic promise, as they can differentiate into all cell types in the body [29,30]. However, research in this domain is constrained by ethical dilemmas surrounding the extraction of stem cells from embryos [19]. iPSCs are anticipated to circumvent these ethical issues while offering utility akin to ESCs. Nonetheless, challenges persist in the reprogramming process, and uncertainties exist regarding their stability [19]. Despite active research and reporting on the therapeutic potential of stem cell therapy, many facets of stem cell biology remain unexplored, including fundamental mechanisms governing stem cell behavior and their interactions with the host environment. Consequently, stem cell therapy has not yet attained widespread adoption as a standard treatment. This review focuses on HSCs and their microenvironment to enhance our understanding of stem cell therapy, especially hematopoietic stem cell therapy.

## 2. Hematopoietic Stem/Progenitor Cells

HSCs are a rare population of multipotent cells, responsible for replenishing all blood cell types throughout an individual’s lifetime. They have the unique ability to self-renew and differentiate into several types of blood and immune cells. This process, which produces all types of blood cells, is called ‘hematopoiesis’ (Figure 1) [9]. HSCs produce hematopoietic progenitor cells through differentiation, which differentiate further to produce blood and immune cells [1]. However, hematopoiesis is a highly regulated process and typically unidirectional; once HSCs differentiate into hematopoietic progenitor cells, they cannot regenerate into HSCs [1]. Additionally, HSCs are used in transplantation therapy after irradiation to treat patients with blood cancer [19]. Unlike solid cancers, which can be selectively targeted and treated, blood cancers present significant challenges for treatment with conventional chemotherapy and radiation. For this reason, HSC transplantation remains one of the most effective and promising approaches, with significant ongoing research focusing on its potential [10].

HSCs predominantly reside in a specialized microenvironment within the bone marrow, known as the endosteum [2,9]. In this niche, HSCs remain dormant under stable conditions. When blood cells decrease due to stressors, such as bleeding, illness, or radiation, HSCs activate and reorganize the hematopoietic system by proliferating and differentiating into new cell types [1]. The equilibrium between the quiescent state and the division of HSCs is crucial for maintaining normal hematopoiesis. If this equilibrium is not adequately regulated, HSCs may decrease in number or give rise to blood malignancies such as leukemia (Figure 1). Thus, the equilibrium between the dormant and active phases of HSCs is tightly controlled by both internal and external mechanisms.

## 3. The Relationship between Hematopoietic Stem/Progenitor Cells and Aging

### 3.1. The Quantity and Role of Hematopoietic Stem/Progenitor Cells in Aging

Blood is an essential regenerating tissue that is susceptible to changes and deterioration with age [12,13,31]. Aging is accompanied by various clinically significant conditions that affect the hematopoietic system [14], including a decline in the adaptive immune system, an increased occurrence of specific autoimmune diseases, a higher prevalence of hematological malignancies, and an increased likelihood of age-related anemia [32]. An age-related decline in the functional capacity of HSCs has been widely recognized in studies conducted on mouse models [33]. When comparing young HSCs to old ones, the latter exhibit a preference for the myeloid lineage and have a reduced ability to regenerate when transplanted [33]. In addition, like many other tissues, the hematopoietic system is more likely to develop cancer with age, including a higher incidence of chronic and acute leukemia [14]. Given that myeloid leukemia is more common in older individuals and juvenile leukemia typically affects the lymphatic system, age-related alterations in HSCs may directly influence the development of disorders associated with blood cell formation [15]. Aged HSCs show increased expressions of genes implicated in the progression of myeloid leukemia, such as *AML*, *PML*, and *ETO*. Alternation of these gene expressions during normal hematopoiesis can result in impaired self-renewal capacity of HSC, heightened susceptibility to DNA damage, and aberrant differentiation potential. These alternations on HSCs are characteristic features of aged HSCs. Consequently, they are deemed suitable targets for investigating HSC aging and comprehending the molecular mechanisms underlying age-related hematopoietic dysfunction and leukemogenesis [32,34,35,36,37,38].

### 3.2. Heterogeneity of Hematopoietic Stem/Progenitor Cell Aging

Multiple studies have documented the deterioration of HSCs in older mice, although the specific molecular processes responsible for this aging phenomenon remain unclear [14,15,31,32,33]. The aging of HSCs is limited by their diversity. The purity of HSCs isolated using flow cytometry has consistently been poor, indicating that the population becomes more heterogeneous as individuals age [15]. Ongoing research aims to identify specific subsets of HSCs that contribute to the aging phenotype [11]. This is achieved through the examination of age-dependent diverse pools of HSCs using single-cell bone marrow transplantation, flow cytometry, and single-cell transcriptome sequencing [15,32,39]. Specifically, HSC clones that undergo myeloid differentiation progressively occupy the HSC reservoir with age [39]. In this aspect, multiple research findings have been reported concerning the correlation between clonal hematopoiesis and aging [40]. Clonal hematopoiesis (CH) is a condition characterized by the expansion of specific HSC clones that acquire somatic mutations (e.g., *DNMT3A*, *TET2*, and *ASXL*) [41,42]. These mutations are thought to confer a selective advantage to HSCs, leading to the predominance of these clones in the blood system and allowing them to outcompete normal HSCs and expand clonally. While the specific signaling pathways involved in this process may vary depending on the gene and context, some common themes have emerged. For example, mutations in *DNMT3A* [43,44], *TET2* [45,46,47], and *ASXL1* [48] are known to affect epigenetic regulation, leading to alterations in gene expression patterns and cellular differentiation pathways [42,49]. Additionally, these mutations may impact other signaling pathways related to cell survival, proliferation, and self-renewal [48]. However, the exact signaling pathways or mechanisms through which these mutations lead to clonal expansion are still under investigation and continue to be an active area of research. This phenomenon becomes increasingly common with age and is associated with a higher risk of hematologic malignancies and cardiovascular diseases [41,50,51]. Research indicates that approximately 10–20% of individuals over 70 years old exhibit clonal hematopoiesis, highlighting its prevalence in the elderly population.

CH not only alters the composition of the hematopoietic system but also impacts the bone marrow microenvironment, known as the niche, which is crucial for maintaining HSC function and homeostasis. Aging induces significant changes in the bone marrow niche, including a decline in the number and function of MSCs, osteoblasts, and endothelial cells [41,52]. These alterations, coupled with the production of elevated levels of inflammatory cytokines such as IL-6 and TNF-α by mutant HSCs and the aging niche, create a pro-inflammatory and oxidative stress environment [47,53,54]. This environment promotes the expansion of CH, impairs normal HSC function, and decreases the secretion of essential factors for HSC maintenance, thus exacerbating the proliferation of clonal HSCs and diminishing the niche’s ability to support normal hematopoiesis. Although there have been numerous reports on the heterogeneity of HSCs associated with aging, our understanding of the effects of aging remains uncertain, and requires further investigation.

### 3.3. Regeneration of Aged/Mature Hematopoietic Stem or Progenitor Cells

A recent study investigated the functional alterations that occur in aged HSCs within the mitochondrial metabolic milieu [12,13,14]. Specifically, the properties and roles of young and aged HSCs are influenced by the mitochondrial membrane potential within these cells [55]. Researchers reversed aging in old mice by manipulating the mitochondrial membrane potential of aged HSCs using the antioxidant Mito-Q [31]. Clinical utilization of Mito-Q is a possible preventative measure and treatment for age-related blood disorders.

## 4. Bone Marrow Microenvironment

HSCs typically reside in the bone marrow (BM), which is composed of various components, including bone, blood vessels, and other cells and substrates filling the spaces between them [2]. This BM microenvironment, known as a “Niche”, provides a structural framework and communication networks to HSCs [2,7].

This microenvironment can control the state of HSCs by direct or indirect interactions and safeguard them from sustaining their undifferentiated state [2,7,9]. It engages HSCs to control their growth and specialization through distinct signal transduction processes, resulting in regular hematopoiesis [7]. Recent advancements in single-cell analysis techniques have revolutionized our understanding of the BM niche, shedding light on its cellular composition, spatial organization, and dynamic interactions with HSCs. One of the key insights gleaned from single-cell analysis is the dynamic nature of the BM niche [56]. Studies have revealed the presence of specialized niches within the BM, each tailored to support specific stages of hematopoietic development [57,58]. Moreover, single-cell analysis has unveiled the plasticity of niche cells, demonstrating their ability to dynamically respond to extrinsic signals and adapt to changing physiological conditions [59,60]. Furthermore, single-cell analysis has provided insights into the spatial organization of the bone marrow niche, uncovering intricate spatial relationships between niche components and HSCs. Spatial transcriptomics techniques have revealed specialized niches localized within specific anatomical regions of the BM, highlighting the importance of spatial context in regulating hematopoietic function [58,61,62,63].

Depending on their spatial location, niches can be divided into an “osteoblastic niche”, which is the area near the endosteum, and a “vascular niche”, where blood vessels and surrounding matrix exist in the BM [58]. In addition, various immune cells derived from HSCs (including T/B lymphocytes, macrophages, natural killer cells, and dendritic cells) or the stromal cells contribute to configuring the BM microenvironment. These cells interact with HSCs, participating in the regulation of their state. Non-cellular substances can also serve as nutrients for HSCs, providing essential support for their growth and maintenance. These substances may include growth factors, cytokines (e.g., SCF, interleukins, CXCL12), and extracellular matrix components present in the BM microenvironment. By interacting with HSCs, these non-cellular factors play a crucial role in regulating hematopoiesis and maintaining stem cell homeostasis.

## 5. Vascular Niche

The vascular niche is composed of endothelial cells and perivascular stromal cells (such as pericytes and smooth muscle cells) that make up blood vessels [64,65,66]. They provide structural support and produce niche factors essential for HSC maintenance, proliferation, and differentiation. Additionally, the extracellular matrix surrounding these niche cells serves as a dynamic scaffold that facilitates cellular interactions and regulates the release and localization of signaling molecules [67,68].

Vasculogenesis can be categorized into two stages: the embryonic and adult stages [2,9]. During the embryonic stage, there is a significant level of contact between HSCs and endothelial cells [69]. Hematopoietic and endothelial cells are derived from hemangioblasts, multipotent progenitor cells, during the embryonic stage [70]. Endothelial cells expressing *RUNX1* can produce HSCs in the aorta, gonad, mesonephros, and placenta [71]. Both endothelial and hematopoietic stem cells co-express *CD31*, *CD34*, *CD133*, *FLK1*, and *TIE2* [72]. HSCs release angiopoietin-1 (ANG1), which stimulates the growth of new blood vessels during angiogenesis [73]. Additionally, endothelial cells provide a similar microenvironment for HSCs as well as neural stem cells. In the hippocampus, neural stem and endothelial cells that generate fibroblast growth factor (FGF), another angiogenesis-promoting substance, are close to each other [74].

However, the precise nature of the interaction between endothelial cells and bone marrow HSCs in the adult stage remains unclear. BM-derived endothelial progenitor cells participate in postnatal angiogenesis [75]. A conceptual framework for the vascular environments in bone marrow has been suggested, wherein the activation of *MMP9* expressed in the osteoblast region results in the separation of the Kit ligand from the cell membrane of stromal cells in the BM. Subsequently, the soluble Kit ligand stimulates the initiation of the cell cycle and enhances the activity of HSCs [76]. Thus, HSC activity, proliferation, and differentiation occur in the vascular niche within the BM [69]. Vascular endothelial growth factor (VEGF) and ANG1 are angiogenic factors that play crucial roles in preserving HSCs [77]. VEGF controls the development of blood vessels and hematopoiesis and regulates hematopoietic stem cells through an internal autocrine loop [78]. HSCs remain inactive in osteoblastic niches, whereas both hematopoietic stem and progenitor cells undergo division in vascular habitats. Hematopoietic cell migration commences in stem cells located in the osteoblast niche where they then proliferate, differentiate, and ultimately mature [7]; cells migrate toward the vascular niche via this process.

To maintain hematopoietic homeostasis, the process of homing, wherein hematopoietic stem and progenitor cells (HSPCs) circulating through the blood return to the BM niche, is also essential [79,80,81]. In this process, HSPCs directly interact with the endothelium via cell–cell adhesive interaction. Sinusoidal endothelial cells express adhesion molecules, including P-selectin (CD62P), E-selectin (CD62E), and vascular cell adhesion molecule-1 (VCAM-1 or CD106). Several receptors for these molecules are expressed in HSPCs, including P-selectin glycoprotein ligand-1 (CD162) and CD44, along with other less well-defined E-selectin receptors. Additionally, receptors for VCAM-1, such as integrins α4β1, α4β7, and α9β1, are also expressed.

The other components such as pericytes and smooth muscle cells also play an important role in regulating the behavior of HSCs [82,83]. Leptin-receptor-positive (LepR^+^) cells and CXCL12-abundant reticular (CAR) [82] cells are well-established cells that secrete growth factors essential for the maintenance of HSCs. They are located along the blood vessels of mainly the sinusoids, playing a crucial role in regulating vascular stability and function. CXCL12 and SCF from them are key factors for HSC proliferation [84]. This was confirmed through experiments deleting CXCL12 secreted by LepR^+^ cells and CAR cells. Deletion of CXCL12 in these cells results in the removal of all quiescent and serially transplantable HSCs from adult bone marrow. This occurs because signaling with CXCR4, receptors on HSCs, is reduced, demonstrating that CXCL12 from LepR^+^ cells and CAR cells play a central role in the signaling that maintains the pool of HSCs [85].

Conversely, Nestin-positive (Nes^+^) cells found exclusively around arterioles provide support, contrasting with perivascular cells around sinusoids [86]. Nes^+^ cells also secrete soluble factors like CXCL12 and SCF, which tend to drive quiescent HSCs into early hematopoietic stages and promote HSC activation, leading to differentiation [87].

## 6. Osteoblastic Niche

Osteoblasts, layering the endosteal bone surface and providing an osteoblastic niche to HSCs, regulate hematopoiesis [7,88]. They provide a supportive environment for HSCs, regulating their self-renewal, differentiation, and quiescence. Osteoblasts produce niche factors and adhesion molecules that interact with HSCs, influencing the maintenance of HSCs in a dormant state and their activation in response to hematopoietic demand [89]. Osteoblasts have a critical role in the regulation of the physical location and proliferation of HSCs by expressing osteopontin (OPN). OPN specifically binds to beta1 integrin expressed on HSCs [90]. The other key factor expressed in osteoblasts is angiopoietin-1 (ANG1). Interaction of Tie2 and ANG1, the receptor of ANG1 expressed on HSCs, vital for maintaining HSCs in the quiescent state, preserves their long-term self-renewal potential and prevents exhaustion [39]. This signaling helps to retain HSCs in the bone marrow niche and prevents their premature differentiation or migration [91,92,93].

Through long-term in vivo labeling with 5-bromodeoxyuridine (BrdU), most HSCs divide [94]. However, some HSCs were found to be dormant, retained their labels, and remained dormant for several months. Therefore, bone marrow cells can be classified into resting and dividing HSCs. Resting HSCs are located close to osteoblasts [7]. Using Bmpr1a KO mice, Zhang et al. showed that N-cadherin^+^ spindle-shaped osteoblasts resemble HSCs with a slow cell cycle [94]. Their study revealed that osteoblast cells expressing N-cadherin in the bone marrow act as nests for HSCs, and that an increase in the number of N-cadherin^+^ cells is associated with an increase in HSCs. Additionally, Visnjic et al. showed that hematopoiesis is suppressed in osteoblast-deficient mice [95]. Thus, it was confirmed that defects in HSC osteoblasts inhibit hematopoiesis. The Notch signaling pathway, characterized by membrane-bound ligands, regulates cell fate determination across various systems, including the self-renewal of HSCs [96,97,98,99,100]. In the study by Calvi et al. [101], they found that PPR-stimulated osteoblasts express a high level of Notch ligand jagged 1 using the transgenic mouse of PTH/PTHrP receptors (PPRs). In response, the activation of the Notch1 intracellular domain (NICD) in Lin-Sca-1+c-Kit+ HSCs increased. Additionally, when HSCs were long-term co-cultured with a Notch cleavage inhibitor, the support for HSCs observed in transgenic stroma decreased to a similar level to their isotype control. Another study, using RAG-1-deficient mice essential for V(D)J recombination and lymphocyte development, showed that Notch1 activation leads to inhibition of HSC differentiation [98]. This confirms that interaction between osteoblasts and HSCs via the Notch pathway plays a crucial role in regulating HSC behavior within the bone marrow niche.

## 7. Other Components of Niche

In addition to spatially distinct osteoblastic and vascular niches, stromal cells and immune cells play roles within the microenvironments of HSCs in bone marrow [62,63,102,103,104]. They can either directly interact with HSCs or regulate them indirectly by secreting soluble factors such as growth factors, cytokines chemokines, and other signaling molecules.

Macrophages in the bone marrow play a crucial role in the formation of HSCs [63,105,106,107,108,109,110,111]. CD169^+^ macrophages, associated with the clearance of blood-borne pathogens and regulation of immune responses, play a crucial role in maintaining the quiescent state of HSCs [105]. They interact with Nestin-positive (Nes^+^) cells to promote the transcription of CXCL12 and other factors (such as HSC maintenance and retention factors ANG, KITl, VCAM1) essential for HSC maintenance. Depletion of macrophages leads to the loss of these factors and subsequent egress of HSCs from the bone marrow [105,106]. A subset of macrophages called ‘Osteomacs’ reside adjacent to osteoblasts and megakaryocytes along the bone lining, distinct from osteoclasts. These osteomas have been identified to play crucial regulatory roles in modulating osteoblast function. Their interaction with osteoblasts is essential for the low-level activation of nuclear factor κB (NF-κB) in osteoblasts, enabling them to maintain HSCs through appropriate chemokine signaling. Furthermore, the presence of megakaryocytes supports the function of osteomacs, and their synergistic interactions with osteoblasts contribute to the regulation of HSC repopulating potential, as evidenced by transplantation assays [107,108,109,110,111]. Although significant progress has been made in understanding the role of macrophages in HSC behavior [106], the specific signaling pathways and the diverse functions associated with macrophage heterogeneity are not yet fully understood. Therefore, ongoing additional studies are needed to fully elucidate the multifaceted roles of macrophages in hematopoiesis and their potential therapeutic applications.

Megakaryocytes also govern the viability of HSCs [112,113,114]. Megakaryocyte removal from the bone marrow leads to an increase in the number of HSCs. HSCs exhibited a compensatory increase in mice experiencing bleeding. However, this compensatory increase is restricted when blood cells are introduced into the bloodstream [113]. Megakaryocytes have been suggested to restrict the proliferation of HSCs in two ways. The first mechanism involves the production of CXCL4 by megakaryocytes, which inhibits HSC proliferation [112]. The second mechanism involves the action of TGFβ, which controls the inactive state of the HSCs [113]. Additionally, megakaryocytes influence myeloid-biased HSC activity and act as a physical barrier to HSC migration. Thrombopoietin (TPO) production by megakaryocytes further regulates hematopoietic activity. Depletion of megakaryocytes in mice resulted in decreased megakaryopoiesis, alongside lower numbers of HSCs and reduced HSC quiescence [115,116,117,118].

Chemokines, also known as chemo-attractant proteins, play crucial roles in regulating the movement of HSCs and facilitating their contact with stromal cells [119]. CXCL12, also known as SDF1, is a chemokine involved in cell homing. Deletion of *SDF1* or its receptor CXCR4 leads to normal fetal heart hematopoiesis; however, there is a failure of bone marrow engraftment by hematopoietic cells [120,121]. Upregulation of CXCR4 in human hematopoietic progenitor cells results in enhanced engraftment in nude mice, whereas the use of CXCR4-neutralizing antibodies demonstrates an inhibitory effect on engraftment [122]. However, CXCR4 is not typically found in HSCs that are not actively dividing. This identifies the factors for successful HSC attachment and the molecules responsible for binding to osteoblasts. Osteoblasts express the adhesion molecules ALCAM and osteopontin, which may play a role in the interaction between HSCs and osteoblasts [123]. Furthermore, it is assumed that external factors such as BMPs, NOTCH ligands, and angiopoietins in bone marrow niches play a role in the interaction between HSCs and osteoblasts [94,101]. In some research, depletion of CXCL12 in osteoblasts resulted in the selective loss of B-lymphoid progenitors. Studies have shown that acute inflammation can inhibit osteoblastic bone formation, leading to T and B lymphopenia due to decreased production of interleukin-7 (IL-7). This suggests that osteoblasts may regulate common lymphoid progenitors by supplying IL-7 [124,125,126].

Myeloid lineage cells, including granulocytes and dendritic cells, also impact the HSC niche [127]. Granulocytes produce factors like G-CSF (granulocyte colony-stimulating factor), which promotes HSC mobilization from the bone marrow into the bloodstream. Dendritic cells contribute to HSC maintenance by modulating the expression of adhesion molecules and cytokines within the niche.

## 8. Stem Cell Therapy

Due to the characteristics of HSCs, their self-renewal, multiple differentiation, and interactions with niche components, they can be used for the therapy of some blood-related diseases. Transplanting HSCs can restore patients’ HSC pools and also regenerate immune cell populations, which means that abnormal hematopoiesis has been replaced with normal hematopoiesis [128]. Hematopoietic stem cell transplantation (HSCT), also known as bone marrow transplantation, is utilized as a therapeutic approach for various blood-related diseases. HSCT offers a powerful therapeutic option by essentially resetting the hematopoietic and immune systems, allowing for the restoration of normal function and providing a potential cure for many serious conditions. It can be applied to patients as a therapeutic approach for various blood-related diseases, including malignant blood disorders such as lymphoma, multiple myeloma, and leukemia, as well as aplastic anemia and immunodeficiency disorders. It is especially considered in relapsed or refractory cases that do not respond to conventional chemotherapy or radiotherapy and in aggressive forms (e.g., diffuse large B-cell lymphoma, mantle cell lymphoma, and follicular lymphoma) [22,129,130,131].

### 8.1. HSC Transplantation

Unlike solid organ transplantation, where the main goal is organ replacement, allogeneic hematopoietic cell transplantation for hematologic malignancies focuses on regulating the immune response against the underlying cancerous condition [128,132]. In leukemia, normal hematopoietic microenvironments are transformed into leukemic microenvironments by leukemic stem cells (LSCs). LSCs exhibit a high propensity for proliferation rather than differentiation into subset populations and possess strong resistance to drugs, resulting in poor prognosis and leukemia relapse [132,133,134,135,136]. For bone marrow transplantation, the most important thing is “donor selection” [137]. It is crucial to match the donor’s human leukocyte antigen (HLA) with the recipient’s as closely as possible to minimize the risk of graft rejection and graft-versus-host disease (GVHD). GVHD is a significant complication following HSCT, where donor immune cells attack the recipient’s tissues, leading to organ damage [138,139]. Immune checkpoint molecules such as TIGIT, PD-1, CTLA-4, and TIM-3 play pivotal roles in regulating immune responses in GVHD [140,141]. TIGIT and PD-1 inhibit T cell activation and effector functions [140,142,143,144,145,146], while CTLA-4 competes with CD28 for ligand binding, thereby inhibiting T cell activation [141,147]. TIM-3 regulates T cell exhaustion and tolerance [148,149]. Dysregulation of these markers can disrupt immune homeostasis, exacerbating GVHD pathology. Understanding the functions of immune checkpoint molecules is crucial for developing targeted therapies to mitigate GVHD severity post-HSCT. In a German study, after transplantation, the graft versus leukemia (GvL) effect in acute myeloid leukemia (AML) was found to significantly improve the 7-year relapse-free survival of patients with AML in first complete remission compared to conventional chemotherapy alone. This highlights its efficacy in disease control. However, transplantation at an advanced disease stage yields lower survival rates, emphasizing the importance of early consideration and referral for transplantation in eligible patients [150,151,152].

### 8.2. Immune Recovery after HSCT

**Figure 3:**
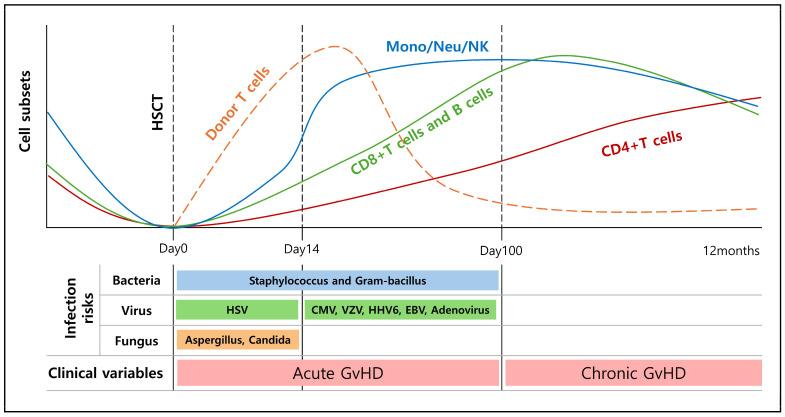
Dynamics of immune reconstitution and associated risks in recipients’ bone marrow following hematopoietic stem cell transplantation. In the first few weeks after transplantation, innate immune cells recover swiftly. Common infections during this phase include bacterial and Candida infections due to the early deficiency in adaptive immune cells. Meanwhile, adaptive immune function, including T cells and B cells, exhibits prolonged deficiencies and gradually recovers, taking over 2 years to fully restore. Viral infections and those caused by non-Candidal molds become more common during this phase. Various clinical factors, including conditioning regimens, donor sources, and post-transplant events such as graft-versus-host disease (GVHD) and immunosuppression, exert influence over the immune reconstitution process, thereby modulating the associated infectious risks.

For successful transplantation, the recipient’s (patient’s) blood and immune system must initially be depleted by combinations of chemotherapy and radiotherapy [153]. Drugs used in conditioning therapy before bone marrow transplantation include cyclophosphamide, busulfan, melphalan, and fludarabine. These drugs induce apoptosis by interfering with DNA replication, transcription, and synthesis, thereby destroying the patient’s existing cells and suppressing the immune system. This helps prevent transplant rejection by adequately suppressing the immune system [154,155,156,157]. If this pre-HCT conditioning is performed well, donor HSCs can home to and engraft the recipient’s bone marrow, thereby reconstituting all the blood cell lineages. Immune recovery after HSCT occurs in phases, with innate immune cells and platelets generally recovering within weeks after HSCT; fully complete reconstitution of adaptive immunity may extend over months to even years (Figure 3) [158,159,160,161].

During this process, various cells within the bone marrow serve as niche components for donor HSCs. The BM niche provides the microenvironment necessary for hematopoietic stem cell (HSC) maintenance, differentiation, and proliferation. Endothelial cells play a significant role in the regulation of various processes, including the quiescence, proliferation, and mobilization of HSCs. It is anticipated that ECs will aid in the hematopoietic recovery of donor HSCs following transplantation. Although ECs are often damaged during conditioning for HCT, when transplanted alongside HSCs, they have been shown to confer beneficial effects in terms of HSC engraftment, reconstitution, and survival post-irradiation [162,163,164]. MSCs, as a rare component of the bone marrow niche, play a crucial role in regulating HSC homeostasis through the production of key soluble factors. Different subsets of MSCs have distinct impacts on HSC behavior, supporting either quiescent or proliferative states. Despite surviving conditioning regimens, MSCs may accumulate damage, potentially affecting their functionality. In clinical contexts, MSCs have shown promise in enhancing HSC engraftment and treating complications like steroid-resistant aGvHD, although further research is needed to elucidate their precise mechanisms of action [165,166,167].

## 9. Conclusions

Hematopoietic stem cells (HSCs) possess the remarkable ability to generate all lower cells of the hematopoietic hierarchy and regulate the entire process of hematopoiesis through self-renewal and proliferation. The uninterrupted generation of new blood cells is indispensable for the survival of organisms, underscoring the critical importance of maintaining the normal function of HSCs throughout life. Normal hematopoiesis involves maintaining a good balance between activated HSCs that produce blood and quiescent HSCs that do not function. However, when HSCs are damaged due to various factors, such as aging, their function is compromised, leading to aberrant hematopoiesis and potentially giving rise to hematological diseases, including aplastic anemia, myelodysplastic syndromes, and leukemia.

Aging affects the overall functioning of an organism, and blood production is also strongly affected. Various research results have revealed that aging affects the function of HSCs, causing their parts to change abnormally. Functional and genomic analysis has been conducted through mouse experiments, and the phenotype in elderly people is similar. Aging eventually causes diseases such as immune disorders, lymphoma, and leukemia, and the prognosis is worse for elderly patients whose hematopoiesis and immune systems have already collapsed.

The niche of HSCs interacts with cells in various aspects to regulate their functions. Osteoblast cells in the bone-adjacent area of the bone marrow lumen play a crucial role in regulating the state of HSCs through various mechanisms. Osteoblasts express Ang1 and OPN, which bind to specific receptors expressed on HSCs, causing them to remain stationary in a specific area. This interaction helps maintain the quiescent state of HSCs and regulates their retention within the bone marrow niche.

Vascular tissue refers to vascular components including vascular endothelial cells, pericytes, and SMCs, as well as stromal cells, which are supporting cells around them. Endothelial cells (ECs) and pericytes are classified according to the location of blood vessels (sinusoids or arterioles), and they both regulate HSCs by secreting various chemokines, including CXCL12 and SCF. These soluble factors perform different functions depending on their site of secretion, either promoting the quiescence or activation of HSCs.

HSC transplantation is gaining attention as a treatment for diseases stemming from HSC damage, particularly leukemia. Just as HSCs interact with niche components to sustain ongoing hematopoiesis, hematopoiesis can be restored by transplanting HSCs from a healthy donor into patients with HSC or niche defects. However, due to the limited understanding of the niche in the context of bone marrow transplantation, ongoing research is crucial to address issues like GVHD.
